# Artificial Intelligence-Driven Dietary Assessment Software: A Structured Narrative Review of Technological Advances, Persistent Measurement Error and Barriers to Clinical Translation

**DOI:** 10.3390/nu18203406

**Published:** 2026-10-17

**Authors:** Mohammad Idreesh Khan, Abrar Turki, Shadaid Alanezi, Malak Ghazi Almutairi, Fauzia Ashfaq, Mohd. Faheem Khan

**Affiliations:** 1 Department of Basic Health Sciences, College of Applied Medical Sciences, Qassim University, P.O. Box 6666, Buraydah 51452, Saudi Arabia; 2 Clinical Nutrition Department, College of Applied Medical Sciences, University of Hafr Al Batin, Hafr Al Batin 39524, Saudi Arabia; 3 Department of Health Information Management and Technology, College of Applied Medical Sciences, University of Hafr Al Batin, Hafr Al Batin 39524, Saudi Arabia; 4 Department of Clinical Nutrition, College of Nursing and Health Sciences, Jazan University, Jazan 45142, Saudi Arabia; 5 Department of Biotechnology and Microbiology, Khandelwal College of Management, Science and Technology, M.J.P. Rohilkhand University, Bareilly 243006, India

**Keywords:** artificial intelligence, dietary assessment, nutrition software, mobile health, computer vision, wearable sensors, precision nutrition

## Abstract

**Background:** Accurate dietary-intake assessment underpins nutrition research and clinical dietetics, yet self-report methods remain vulnerable to recall, portion-size and reporting error. **Objective:** This structured narrative review evaluates AI-enabled and sensor-supported dietary-assessment software, focusing on technical performance, measurement validity, user engagement, food-composition databases, cultural applicability and clinical readiness. **Methods:** Evidence was identified through searches of five electronic sources—PubMed (including MEDLINE-indexed records), Scopus, Web of Science Core Collection, IEEE Xplore and ScienceDirect—covering January 2020 to March 2025, and a complete rerun of the same five sources on 7 August 2026, supplemented by backward and forward citation tracking. The retained primary screening record comprised 1620 electronic-source records and 42 additional records (1662 before deduplication); 478 duplicates were removed, 1184 records were screened, 212 full texts were assessed and 15 sources were retained for the primary-phase qualitative synthesis. These figures are retained aggregate review-log totals and should not be interpreted as a fully reconstructable systematic-review audit trail. The 2026 update incorporated 18 publications: 10 from the January 2020–March 2025 historical interval through separate recovery/reassessment, and eight published after March 2025 through the 497-record update-specific screening stream, with retrieval phase reported separately from evidence role. The higher, moderate and limited/emerging within-map convergence descriptors summarise patterns in the purposively selected literature and do not represent exhaustive retrieval, formal certainty of evidence or reproducible screening. Results: Food recognition is most mature for visually distinct foods under controlled conditions. Portion-size estimation, nutrient quantification, mixed dishes, sustained engagement and culturally diverse foods remain less consistent. Selected systems show reasonable group-level energy and macronutrient agreement under defined conditions, whereas validation in free-living and clinical settings remains variable; a 2026 doubly labelled water study reported substantial underestimation and poor individual agreement in females with obesity. **Conclusions:** Current tools may support self-monitoring, research screening and dietitian-assisted assessment, but stand-alone use for high-stakes therapeutic decisions requires independent, nutrient-specific validation in the target population.

## 1. Introduction

Accurate quantification of food intake remains essential for nutrition research, clinical dietetics and public health policy. Major non-communicable diseases, including obesity, type 2 diabetes mellitus, cardiovascular disease, osteoporosis and micronutrient deficiency, are strongly influenced by dietary patterns. Dietary data therefore underpin epidemiological surveillance, clinical decision-making and the evaluation of nutrition interventions. Despite decades of methodological development, measuring diet accurately in free-living settings remains one of the most persistent challenges in nutrition science [1]. Conventional dietary assessment methods, including 24 h recalls, food frequency questionnaires (FFQs) and multi-day food records, have been the principal tools in nutrition research for decades. These methods are transparent and well characterised, but they rely on memory, self-reporting and subjective estimation of portion size. Consequently, they are vulnerable to recall bias, social-desirability bias and systematic misreporting [1,2]. Misreporting is especially problematic in populations living with overweight or obesity, where underreporting of total energy intake is frequently observed [1,2]. Repeated recalls and detailed food diaries can also increase respondent burden, reduce compliance and contribute to incomplete longitudinal datasets [1]. Although trained interviewers and standardised probes can reduce some errors, they increase cost, logistical complexity and scalability challenges in large studies [3]. These limitations have driven the search for dietary assessment approaches that are less burdensome and more accurate in real-world settings.

The global adoption of smartphones and mobile health (mHealth) platforms has transformed dietary assessment. Nutrition applications now allow users to log meals, estimate nutrient intake, scan barcodes and receive tailored dietary feedback in near-real time [4,5]. More recently, advances in artificial intelligence (AI), machine learning and computer vision have accelerated the development of image-based and automated dietary assessment systems. These systems aim to identify foods and estimate portion sizes from smartphone images, thereby reducing reliance on memory and manual data entry [6]. In parallel, wearable sensors use motion, acoustic or physiological signals to detect eating events and can be integrated with activity, behavioural and clinical data [7,8]. In principle, these technologies could shift dietary assessment from retrospective, episodic reporting toward more continuous and context-rich monitoring, with potential benefits for research and clinical care [9].

Despite rapid technical progress, digital dietary tools have not fully resolved the core measurement problems they were designed to address. Validation evidence indicates tool-dependent agreement for total energy and macronutrient estimates, while micronutrient and usual-intake estimates are less consistent [10]. Mixed dishes, overlapping foods and visually hidden ingredients, such as oils, sauces and sugars, remain important sources of error because they are difficult to infer from surface images alone [6,11,12]. Performance also declines under ecological variability, including changes in lighting, camera angle, image resolution and background clutter [6,11]. Food-recognition models and linked databases may also perform unevenly for culturally diverse foods and preparation methods; direct cross-cultural validation remains limited, although evidence from under-represented populations demonstrates the importance of this issue [13]. These limitations raise concerns about fairness, generalisability and usefulness in global nutrition research and public health programmes.

Technical performance alone does not determine the utility of dietary software. Real-world effectiveness depends heavily on sustained user engagement. Reviews and implementation studies describe strong initial uptake, followed by declining use in many dietary-tracking applications; however, the timing and magnitude of attrition vary across platforms and populations [14,15,16,17]. Even when AI assistance is available, users may still need to correct misidentified foods, adjust portion sizes or manually enter missing ingredients. These tasks reintroduce cognitive and time burdens and can contribute to app fatigue [14,15]. Behaviour-change features such as reminders, gamification and social support may improve short-term engagement, but evidence for durable effects remains inconsistent [15,17]. High-frequency digital data capture can therefore create an impression of methodological superiority without necessarily improving the completeness or validity of dietary information.

For dietary software to move beyond consumer wellness applications into clinical nutrition practice, it must meet higher standards of transparency, accountability and safety. Clinicians require interpretable outputs and auditable methods before using AI-derived estimates to guide dietary treatment. Many systems provide nutrient summaries or recommendations without clearly explaining how foods were identified, portions estimated or nutrient values assigned [18,19]. This opacity can reduce professional trust and complicate clinical accountability. Integration with wearable devices, electronic health records and precision-nutrition frameworks also increases concerns about privacy, data security, interoperability and governance [18,20,21]. Dietary data may reveal sensitive information about health status, socioeconomic circumstances, and cultural or religious practices. Inconsistent data standards and unclear data-sharing policies remain important barriers to institutional adoption.

Research on digital dietary assessment has expanded rapidly, but the literature remains fragmented across technical validation, user behaviour, database quality, cultural applicability and clinical implementation. The review question was as follows: what technical, behavioural, database-related and clinical barriers limit the accuracy, equity and clinical translation of AI-enabled dietary-assessment software? This review synthesises direct evidence, distinguishes it from contextual and methodological sources and identifies priorities for more accurate, equitable and clinically useful dietary-assessment systems.

## 2. Methods

This study used a structured narrative review with purposive evidence selection to evaluate recent evidence on dietary software applications and their limitations. Narrative synthesis was chosen because the evidence spans heterogeneous technologies, populations, outcomes and validation methods, making formal quantitative pooling inappropriate. This review was not prospectively registered as a systematic review and does not claim exhaustive retrieval or full PRISMA compliance. SWiM guidance and PRISMA-S principles were used only as reporting aids for non-pooled synthesis and transparent search description [22,23]. The retained primary review log records 15 sources in aggregate for the primary phase. In the current named bibliographic map, 14 entries can be securely assigned to that primary-phase core set; Cofre et al. [24], first published online on 10 April 2025, is treated separately as a later post-primary secondary-synthesis addition. The 2026 update contributed 18 publications (10 historical-period and 8 post-March-2025 publications). Retrieval phase was recorded separately from evidence role, with cited sources classified as direct, contextual or methodological.

Within this design, a “primary-phase core synthesis source” refers to one of the 14 currently named publications that can be securely assigned to the primary search ending in March 2025. The retained aggregate review log nevertheless records 15 primary-phase sources; because the available record does not identify the unmatched fifteenth source, no identity or route was retrospectively reconstructed. Cofre et al. [24] is classified separately as a later post-primary secondary-synthesis addition because its publisher-recorded first online publication date is 10 April 2025; the exact date and route by which it was later incorporated are not preserved. A “2026-update source” refers to a publication incorporated during the 7 August 2026 update, either through separate historical-period recovery/reassessment (10 publications; [25,26,27,28,29,30,31,32,33,34]) or through the post-March-2025 update-specific screening stream (8 publications; [35,36,37,38,39,40,41,42]). These retrieval-set labels are distinct from evidence role: direct sources inform one or more principal review conclusions, whereas contextual and methodological sources serve the functions described below. Contextual sources define established concepts, describe broader implementation frameworks or interpret implications that were not primary outcomes of direct dietary-software studies. Methodological sources are restricted to reporting and synthesis procedures. This classification reduces the risk that general digital-health or guideline literature will be interpreted as direct empirical evidence of dietary-software accuracy. When original validation studies and secondary reviews addressed overlapping evidence, greater interpretive weight was given to direct validation against a stated reference method; reviews were used to assess convergence, heterogeneity and remaining gaps rather than counted as independent replication.

This review was structured around the following question: what technical, behavioural, database-related and clinical barriers limit the accuracy, equity and clinical translation of AI-enabled dietary-assessment software? A PICOS-equivalent framework was used to define scope. The population and context included users, patients and free-living populations using digital dietary assessment. The intervention or technology comprised AI-enabled, image-based, mobile, wearable or hybrid dietary-assessment tools. Comparators or reference standards included conventional dietary-assessment methods, weighed food records, interviewer-administered recalls, ground-truth images or implementation benchmarks. Outcomes included food recognition, portion-size estimation, nutrient accuracy, user engagement, database quality, interoperability, interpretability, privacy and clinical readiness. Eligible designs included original validation, usability and implementation studies, together with systematic or scoping reviews that directly addressed the review question.

### 2.1. Literature Search Strategy

**Figure 1:**
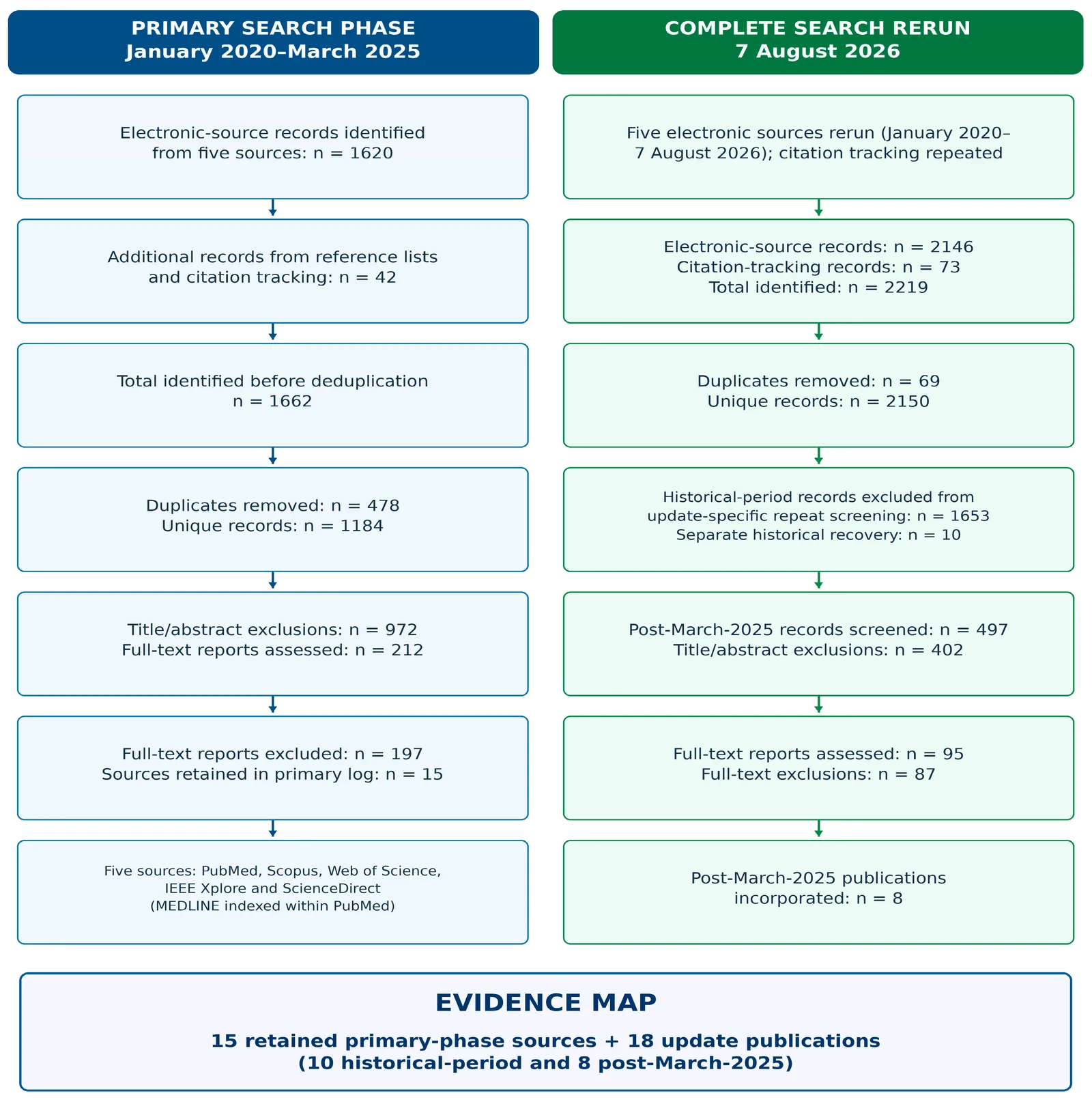
Best-available aggregate reconstruction of the literature identification and evidence-mapping workflow from the retained review log. The primary-phase totals are historical aggregate review-log counts and should not be interpreted as a fully reconstructable PRISMA audit trail because database-specific allocation, exact historical execution dates and itemised reasons for all 197 full-text exclusions were not retained at record level. The 7 August 2026 rerun yielded 2150 unique records after deduplication. Of 1653 historical-period records removed from update-specific repeat screening, 10 publications [25,26,27,28,29,30,31,32,33,34] were recovered/reassessed separately; their current source/index locations are documented in Supplementary Table S1B, but their original update-entry routes were not retrospectively assigned. The 497 post-March-2025 records underwent title/abstract screening (402 excluded); 95 full-text reports were assessed, 87 were excluded and 8 publications were incorporated [35,36,37,38,39,40,41,42], giving 18 update publications in total. MEDLINE-indexed records were retrieved within PubMed. The figure is descriptive of the retained review records and should not be interpreted as demonstrating exhaustive retrieval or fully reproducible historical study selection. The primary n = 15 is retained as an aggregate historical log count; the current named bibliographic mapping can securely assign 14 currently named sources to that phase, while Cofre et al. [24] is a later post-primary addition and is not counted within that named primary set.

The primary search phase covered peer-reviewed literature published from January 2020 to March 2025 and used five electronic sources: PubMed (including MEDLINE-indexed records), Scopus, Web of Science Core Collection, IEEE Xplore and ScienceDirect. MEDLINE was not searched separately; MEDLINE-indexed literature was retrieved within the single PubMed search. The retained review log recorded 1620 electronic-source records and 42 additional records identified through reference-list checking and citation tracking, yielding 1662 records before deduplication. After 478 duplicates were removed, 1184 records underwent title/abstract screening; 972 were excluded, 212 reports underwent full-text assessment, 197 were excluded and 15 sources were retained for the primary-phase qualitative synthesis. These historical counts are reported as retained aggregate review-log totals rather than as a fully reconstructable PRISMA audit trail because database-by-database allocation of the 1620 records, exact historical execution dates and a reason-specific record-level disposition log for the 197 full-text exclusions were not retained in reconstructable form. Figure 1 therefore labels the primary flow as a best-available aggregate reconstruction from the retained review log. Publisher verification shows that Cofre et al. [25] was first published online on 10 April 2025, after the March 2025 primary-search endpoint. It is therefore not assigned to the named primary-phase set and is recorded as a later post-primary secondary-synthesis addition; the retained records do not establish its exact later incorporation date or route. The historical aggregate count of 15 primary-phase retained sources is preserved rather than retrospectively reconstructed. A present-day rerun was not used to overwrite the historical totals because database contents and indexing change over time; such a rerun would constitute a new search rather than a faithful reconstruction of the original historical result set. To ensure currency, the same five electronic sources were rerun on 7 August 2026 across January 2020 through 7 August 2026, using the preserved source-specific expressions, and backward/forward citation tracking was repeated. The rerun identified 2146 electronic-source records and 73 citation-tracking records (2219 before deduplication); after 69 duplicates were removed, 2150 unique records remained. For update-specific repeat screening, 1653 records from January 2020 to March 2025 were removed from the post-March-2025 screening stream, leaving 497 records; 402 were excluded at title/abstract stage, 95 reports underwent full-text assessment, 87 were excluded and 8 post-March-2025 publications were incorporated [35,36,37,38,39,40,41,42]. Separately, 10 historical-period publications [25,26,27,28,29,30,31,32,33,34] were recovered/reassessed during the full-range update, giving 18 update publications in total. The retained records do not permit reconstruction of the original study-level route by which these 10 historical-period publications entered the evidence map. Supplementary Table S1B documents each publication’s DOI and currently verifiable source/index location; these data establish current retrievability, but not the original historical route of identification. Accordingly, the unreconstructed entry pathway is considered a potential source of selection bias. EndNote (Clarivate, London, UK) was used for record management and deduplication. Publisher/index checks were used for bibliographic verification rather than as a separate screening source. Search expressions, field scopes, search-time limits and post-search eligibility restrictions are reported in Supplementary Table S1; the update flow is reported in Supplementary Table S1A. In PubMed, “peer-reviewed” was a post-search eligibility criterion rather than a database filter. The recorded PubMed expression is reproduced verbatim in Supplementary Table S1 and contains “app*”; under current PubMed syntax, truncation requires at least four characters before the asterisk, so this term is reported as a reproducibility limitation rather than silently corrected.

### 2.2. Rationale for Study Selection

Eligibility criteria were defined to identify sources that directly informed the review question and represented contemporary advances in dietary-assessment technology. At title/abstract or full-text stage, records were excluded when they lacked a dietary-assessment focus; did not evaluate a relevant AI, image-based, mobile, wearable or hybrid technology; did not report an eligible outcome such as accuracy, validity, usability, engagement, nutrient estimation, implementation or clinical relevance; were non-peer-reviewed or methodologically opaque; or were editorials, comments, letters, protocols or corrections without usable evidence for the review question. Duplicate reports were consolidated. Because the purpose was structured narrative synthesis rather than exhaustive effect estimation, priority was given to publications with sufficiently transparent information on technical performance, measurement validity, usability, clinical relevance or implementation. The operational inclusion and exclusion framework is summarised in Supplementary Table S1C.

### 2.3. Inclusion Criteria

Sources were eligible for the direct evidence set when they met all of the following criteria: (i) publication in a peer-reviewed source, including original validation research, controlled or comparative studies, systematic reviews or scoping reviews; (ii) direct focus on digital, AI-driven, image-based, mobile or sensor-supported dietary assessment; (iii) evaluation of at least one domain relevant to the review question, including accuracy, reliability, user engagement, adherence, food identification, portion estimation, nutrient estimation, database functionality, clinical relevance or implementation; and (iv) publication mainly between 2020 and 7 August 2026. Only English-language publications were assessed. Foundational publications outside the main interval were retained only as contextual evidence when necessary to explain established methods or concepts.

### 2.4. Exclusion Criteria

Sources were not used as direct evidence when they focused solely on conventional paper-based assessment, evaluated nutrition education without assessing dietary intake, lacked peer-reviewed or methodologically transparent evidence or did not directly inform the review question. Conference abstracts, editorials and commentaries were excluded from the direct evidence set when they did not provide sufficient methodological detail or validated findings.

### 2.5. Study Selection Process

Records identified through electronic-source searching and citation tracking were managed in EndNote. Duplicate records were removed before titles and abstracts were screened against the predefined eligibility criteria, followed by full-text assessment of potentially relevant reports. The retained primary screening record comprised 1662 records before deduplication, including 1620 electronic-source records and 42 records identified through reference lists and citation tracking. Removal of 478 duplicates resulted in 1184 records for title/abstract screening; 972 were excluded, 212 reports underwent full-text assessment, 197 were excluded after full-text assessment and 15 sources were retained for the primary-phase qualitative evidence synthesis. All six authors (M.I.K., A.T., S.A., M.G.A., F.A. and M.F.K.) participated in title/abstract screening, full-text assessment, data extraction, structured evidence/quality appraisal and narrative synthesis. All authors reviewed the eligible literature, and study relevance, extracted evidence, evidence-role assignments and quality classifications were discussed and consolidated by consensus. The workflow was collaborative rather than formal dual-independent screening. Independent duplicate screening was not part of the review design. No separate independent duplicate screening exercise was conducted. A collective or consensus re-review is not equivalent to independent duplicate screening and cannot establish screening reproducibility. Accordingly, reproducibility of the original eligibility decisions cannot be verified. The complete five-source rerun on 7 August 2026 used the same eligibility and evidence-role criteria, with backward/forward citation tracking repeated. The update was reported in two pathways: 497 post-March-2025 records underwent title/abstract and full-text screening (402 title/abstract exclusions, 95 full texts assessed, 87 full-text exclusions and 8 publications incorporated; [35,36,37,38,39,40,41,42], whereas 10 historical-period publications [25,26,27,28,29,30,31,32,33,34] were recovered/reassessed separately during the full-range update. The original study-level route for those 10 publications was not preserved; Supplementary Table S1B therefore documents their currently verifiable source/index locations without presenting those locations as reconstructed historical routes. Update-stage counts are reported in Supplementary Table S1A, evidence roles and reasons for classification in Supplementary Table S2, and the overall evidence-mapping workflow in Table 1 and Figure 1.

The evidence map prioritised publications with verifiable bibliographic records and a defined contribution to a principal conclusion. Direct validation studies, secondary reviews, usability studies and broader implementation literature were not treated as interchangeable. Supplementary Table S2 classifies cited sources as direct, contextual or methodological and explains their evidence role. Supplementary Table S3 links the principal conclusions food recognition, portion estimation, nutrient accuracy, engagement, cultural applicability and clinical readiness to contributing sources, study design, directness, reference standard, limitations, evidence overlap and final within-map convergence descriptor. Original studies represented within secondary reviews were not counted as independent confirmations. Supplementary Table S4 provides a study-level quantitative validation summary for the principal original validation studies, including population or test set, sample size, reference method, key accuracy or agreement results and verified overlap with Cofre et al. [25].

### 2.6. Data Extraction and Evidence Mapping

A standardised evidence-mapping framework was developed to support comparison across direct evidence sources. Extracted variables included author and year, source type, population or setting, type of dietary software, underlying technology, dietary outcomes, validation method or reference standard, user engagement and adherence metrics, and reported limitations or biases. Contextual and methodological publications were recorded separately so that they did not appear to provide direct validation of dietary-software performance. All six authors contributed to data extraction and evidence mapping. Extracted information and evidence-mapping entries were cross-checked within the author team, with differences resolved by consensus, as described in Section 2.5. The principal original quantitative validation studies are tabulated separately in Supplementary Table S4 so that populations or test sets, sample sizes, reference methods, key accuracy/agreement results and verified overlap with secondary synthesis can be traced directly.

### 2.7. Risk of Bias and Quality Considerations

Because the direct evidence set included original validation studies, technical studies, usability research and secondary reviews, no single formal risk-of-bias instrument was suitable for all study designs. Each source was therefore appraised using a structured quality-consideration framework covering appropriateness of the reference standard, directness of evidence, sample size and participant diversity, transparency of AI training and validation, duration of adherence assessment, clarity of food-composition database reporting and explicit discussion of error sources. For internal narrative appraisal, principal conclusions were assigned one of three within-map convergence descriptors: higher convergence, moderate convergence or limited/emerging evidence. These descriptors summarise patterns within the purposively selected evidence map and do not represent completeness of retrieval, formal certainty of evidence, design-specific risk-of-bias judgments or reproducibility of screening. The conclusion-level appraisal is reported in Supplementary Table S3. All six authors participated in the structured evidence/quality appraisal, and the resulting descriptors were cross-checked by the author team and finalised by consensus, as described in Section 2.5.

The within-map convergence descriptors were assigned using four considerations: directness of the source to the stated claim; convergence across independent publications; adequacy of the validation or reference standard; and the extent to which limitations restricted transfer to free-living or clinical settings. Operationally, higher within-map convergence required at least three independent directly relevant publications to converge, including at least one original validation/technical study, with no major contradictory direct evidence identified. Moderate within-map convergence required at least two independent directly relevant sources, or a high-level synthesis corroborated by at least one independent direct study, but with important heterogeneity by tool, population, food type or validation design. Evidence was described as limited/emerging within the map when fewer than two independent direct studies were available, when the evidence was mainly contextual or secondary, or when clinically relevant outcomes had not been evaluated. Secondary reviews that contained overlapping primary studies were treated as synthesis and were not counted as separate empirical replications. These thresholds are pragmatic narrative heuristics applied only to the selected evidence map; they are not formal certainty grades and do not establish exhaustive retrieval or reproducible historical study selection. The currently mapped synthesis sources shown in Table 2 comprise 14 named primary-phase core sources plus the later post-primary Cofre et al. [25] secondary addition; the conclusion-level application is shown in Supplementary Table S3.

**Table 2:** Fourteen named primary-phase core synthesis sources plus one later post-primary secondary addition used in the current structured narrative synthesis. Cofre et al. [24] was first published online on 10 April 2025 and is therefore not assigned to the primary search ending in March 2025; the exact date and route of its later incorporation are not preserved. The retained primary review log still records 15 sources in aggregate, and no missing historical source identity has been retrospectively invented. Supplementary Table S2 classifies cited sources independently by evidence role as direct, contextual or methodological.

Synthesis Source	Source Type/Domain	Evidence Role and With-in-Map Convergence	Quality Considerations
Lo et al. [11]	Technical review; image recognition and volume estimation	Technical limitations; con-tributes to synthesis of a higher-convergence with-in-map conclusion but is not counted as independ-ent primary replication.	Heterogeneous datasets and reference standards.
Dalakleidi et al. [12]	Systematic review; image-based dietary assessment	Recognition, mixed-dish and portion-estimation limitations; contributes to synthesis of a higher-convergence within-map conclusion but is not counted as independent primary replication.	Substantial variation in tools and validation de-signs.
Cofre et al. [24] (later post-primary secondary addi-tion)	Systematic review; AI die-tary-intake validity	Validity and measure-ment-error interpretation; contributes to moderate within-map convergence as secondary synthesis ra-ther than independent primary replication.	Tool-level accuracy and reference methods varied.
Tahir and Loo [43]	Technical survey; recogni-tion and volume estima-tion	Algorithmic performance and environmental limita-tions; contributes to syn-thesis of a higher-convergence within-map conclusion but is not counted as independent primary replication.	Predominantly technical outcomes; limited clinical validation.
Zheng et al. [44]	Scoping review; food and nutrient intake measure-ment	Field-wide capabilities and evidence gaps; contributes to moderate within-map convergence.	Broad scope and heteroge-neous source designs.
Chotwanvirat et al. [45]	Scoping review; food-image assessment	Image-based strengths, limitations and implemen-tation; contributes to mod-erate within-map conver-gence.	Evidence includes diverse technologies and end-points.
Zhang et al. [10]	Systematic review and me-ta-analysis; dietary-record apps	Energy and macronutrient validity; contributes to moderate, tool-dependent within-map convergence.	Application versions and recording methods dif-fered.
Gioia et al. [16]	Evaluation; mobile apps for research use	Usability, data complete-ness and research suitabil-ity; contributes to moder-ate within-map conver-gence.	Rapid software change may affect current ap-plicability.
König et al. [15]	Systematic review; nutri-tion-app barriers	User engagement and im-plementation barriers; con-tributes to moderate with-in-map convergence.	Predominantly self-reported behavioural out-comes.
Turner-McGrievy et al. [14]	Review/perspective; moni-toring burden	Correction burden and engagement; contributes to moderate within-map convergence.	Not a pooled effectiveness analysis.
Bell et al. [46]	Scoping review; wearable eating detection	Eating-event detection and false-positive limitations; contributes to moderate within-map convergence.	Food type and quantity generally unresolved.
Sempionatto et al. [47]	Review; wearable/mobile personalised nutrition	Sensor integration and personalised-nutrition op-portunities; contributes to limited/emerging within-map convergence.	Clinical-outcome valida-tion remains limited.
Wang et al. [48]	Scoping review; sensor-based eating capture	Real-time eating-event capture and workflow in-tegration; contributes to moderate within-map convergence.	Heterogeneous devices and free-living validation.
Abeltino et al. [18]	State-of-the-Art review; diet monitoring and preci-sion nutrition	Clinical integration, data-base and governance con-text; contributes to lim-ited/emerging within-map convergence.	Predominantly descriptive evidence.
Sosa-Holwerda et al. [49]	Scoping review; AI in nu-trition research	Broader translation, equity and research gaps; con-tributes to lim-ited/emerging within-map evidence.	Wide scope; limited direct evidence for specific clini-cal uses.

The named synthesis set shown in Table 2 is heterogeneous and developmental rather than exhaustive. Fourteen listed sources can be assigned to the primary-phase core set, while Cofre et al. [25] is retained as a later post-primary secondary addition. The aggregate primary review log records 15 retained sources, but the identity of the unmatched fifteenth historical source is not reconstructable from the available record and has not been replaced retrospectively. Evidence incorporated during the 2026 update broadened the evidence map with validation, usability and multimodal-AI studies without treating every citation as independent confirmation. Direct validation evidence, secondary synthesis, clinical-outcome evidence and long-term adherence evidence were considered separately. The results use only the higher, moderate or limited/emerging within-map convergence descriptors; these describe convergence within the purposively selected map and do not represent completeness of retrieval, formal certainty of evidence or screening reproducibility.

### 2.8. Thematic Narrative Synthesis

The evidence was synthesised thematically to account for the fragmented nature of the field. Studies were organised into five interrelated analytical themes: (i) technical capabilities and limitations of AI-based dietary assessment; (ii) user engagement, acceptance and behavioural barriers; (iii) food-composition databases’ quality and nutrient accuracy; (iv) clinical integration, interpretability and professional trust; and (v) emerging developments in wearable sensing and precision nutrition. This structure allowed comparison across platforms while preserving the broader scientific and implementation context.

### 2.9. Methodological Rationale

A structured narrative synthesis was used to avoid overstating precision across heterogeneous technologies and validation designs. Sources differed in reference standards, follow-up duration, target populations, software maturity and endpoints. The objective was therefore to identify recurring patterns, methodological gaps and implementation barriers rather than to generate pooled estimates that could imply inappropriate comparability. This approach is suitable for complex digital-health evidence in which performance depends on algorithmic accuracy, user behaviour, data infrastructure, ethics and implementation context [20].

### 2.10. Development of the Proposed Clinical-Readiness Framework

The use-case framework was developed by the author team through consensus interpretation of the mapped evidence. Development considered the directness and consistency of validation evidence, the reference method, the target population, the consequence of error and the availability of professional review. The framework is intended to support future validation and implementation decisions; it is not a regulatory standard, formal consensus guideline or empirically derived set of cut-offs. Current evidence-based readiness and author-recommended validation standards are presented separately.

### 2.11. Strengths and Limitations of the Review Approach

A major strength of this review is its integrative scope, which brings together technical, behavioural, database-related and clinical perspectives within one analytical framework. Important limitations remain. This review is a structured narrative synthesis with purposive evidence selection rather than a prospectively registered systematic review; it does not claim exhaustive retrieval, formal dual-independent screening, design-specific risk-of-bias assessment or GRADE certainty grading. The retained primary review log preserves aggregate identification and screening totals, but not complete source-by-source historical allocations, exact historical execution dates or a fully itemised record-level reason log for the 197 historical full-text exclusions; Figure 1 therefore presents a best-available aggregate reconstruction rather than a fully auditable PRISMA record. The literature was reviewed collaboratively by all six authors and consolidated by consensus. Independent duplicate screening was not undertaken; consequently, reproducibility of the original eligibility decisions cannot be verified. The original study-level entry routes for the 10 historical-period publications [25,26,27,28,29,30,31,32,33,34] were also not preserved; their DOI and current source/index retrievability are documented in Supplementary Table S1B, but these current locations do not reconstruct the historical selection routes. These two limitations may have introduced selection bias by making it impossible to determine whether all eligible records were handled consistently or whether particular routes preferentially contributed to the mapped evidence; the conclusions should therefore be interpreted as patterns within a purposively selected literature map rather than as exhaustive or reproducibly screened evidence. Embase and CINAHL were not searched, which may have reduced coverage of clinically oriented nutrition, nursing and allied-health literature. The English-language restriction may also have preferentially captured tools and validation studies reported in English-language or internationally indexed journals, potentially under-representing local-language applications and food databases developed in Asian, European and other non-English-speaking markets; this limitation may particularly affect conclusions about cultural coverage, database completeness and generalisability. Publication bias may have favoured positive findings, and software versions may have changed after publication. Direct, contextual and methodological evidence were therefore separated, and principal conclusions were appraised at the conclusion level. The recorded PubMed query contains “app*”; under current PubMed syntax, truncation requires at least four characters before the asterisk, so this term remains a disclosed reproducibility limitation of the recorded expression.

## 3. Results

### 3.1. Technical Capabilities and Limitations of AI-Based Dietary Assessment

AI-based dietary assessment systems, particularly image-based and multimodal tools, are widely studied in digital nutrition. Evidence patterns in the Results are described using the higher, moderate and limited/emerging within-map convergence descriptors defined in Section 2.7; these are descriptive categories applied to the purposively selected evidence map, not estimates of exhaustive literature-wide certainty, retrieval completeness or screening reproducibility.

Direct evidence is distinguished from contextual and methodological sources throughout the synthesis, and contextual sources are not treated as independent empirical confirmation. To make update provenance visible at the point of interpretation, citations from the 2026 update [25,26,27,28,29,30,31,32,33,34,35,36,37,38,39,40,41,42] are separated from the earlier/core/contextual literature in key Results statements and in Table 3 and Table 4; detailed source roles remain available in Supplementary Table S2.

#### 3.1.1. Food Recognition Performance

Earlier/core evidence indicates that food-recognition algorithms perform best for visually distinct, single-component foods under controlled conditions [6,11,12,24,43,44,45], and 2026-update publications reinforce this task-dependent pattern [30,32,34,35,36,38,39]. Under the operational criteria in Section 2.7, the selected evidence shows higher within-map convergence for basic recognition tasks but only moderate within-map convergence for complete free-living dietary assessment. Performance declines for mixed dishes, overlapping foods and visually ambiguous meals in earlier/core evidence [11,12,24,43,44,45,50], and in update publications [30,35,38,39].

#### 3.1.2. Portion-Size Estimation and Depth Limitations

Portion-size estimation remains one of the most consistently reported technical weaknesses of image-based dietary software. Earlier/core evidence identifies this limitation [6,11,12,24,43,44,45], and 2026-update studies provide additional direct and technical support [28,30,32,33,34,36,39]. Recognition is primarily a classification task, whereas portion estimation requires conversion of two-dimensional images into three-dimensional volume or mass. Controlled studies and 3D-scaling approaches show technical progress, but estimates remain sensitive to camera angle, distance, framing, food geometry and the availability of a scale reference. The existence of the limitation shows higher within-map convergence; its magnitude remains strongly system-, population- and reference-standard dependent.

#### 3.1.3. The Hidden-Ingredient Problem

Direct evidence consistently identifies the hidden-ingredient problem. Oils, butter, sauces, sugars and cooking fats can contribute substantially to intake but may be absorbed into foods or remain visually indistinguishable [6,11,12,39,45,50]. Because surface images provide only partial information about composition, a system may recognise the visible food while underestimating energy, fat, sodium or added sugar. Multimodal prompts and user confirmation can reduce selected errors but do not eliminate the structural limitation of image-only assessment.

#### 3.1.4. Environmental and Device-Related Variability

Recognition performance is sensitive to lighting, glare, shadowing, background complexity, image resolution, camera angle and device characteristics in earlier/core evidence [6,11,12,24,45] and in 2026-update studies [34,35,39]. These environmental effects show higher within-map convergence for technical recognition tasks. Direct cross-cultural validation remains limited. Earlier/regional evidence [13,44,45,49] and 2026-update studies [31,34,36,39,40] indicate that culturally diverse foods and preparation methods require dedicated datasets and independent validation. Cultural applicability therefore shows limited/emerging within-map convergence; the magnitude of cross-cultural performance differences is not universally quantified.

#### 3.1.5. Computational and Deployment Constraints

High-performing AI systems may rely on cloud-based processing, proprietary foundation models or complex computational pipelines. Earlier/contextual evidence identifies connectivity, privacy, governance, version-control and auditability concerns [18,19,20,44,45], while 2026-update multimodal studies illustrate the growing use of externally hosted model pipelines [34,39]. Validation of consumer and clinical applications in the update evidence also shows that technical sophistication can coexist with substantial measurement error [41,42]. Current evidence therefore supports broad pattern monitoring more strongly than high-stakes nutrient estimation. The principal technology categories are summarised in Table 3.

**Table 3:** AI-based and digital dietary assessment tools: technologies, strengths, limitations and supporting references.

Category of Dietary Software	Core Technology	Main Strengths	Key Limitations	Key Supporting References
Image-based dietary assessment systems	Computer vision, convolutional neural networks (CNNs), deep learning	Reduces recall bias; supports user-friendly food-image capture; enables automated food identification	Portion-size and hidden-ingredient error; sensitivity to image conditions; mixed-dish limitations; model/version dependence.	Earlier/core/contextual: [6,11,12,24,25,26,27]; 2026 update: [42,44,46,47,48,49,51,52,53]
Mobile health (mHealth) dietary applications	Manual food entry, barcode scanning and rule-based nutrient databases	Widely accessible and low cost; large food databases; suitable for short-term energy tracking	Selective logging, correction burden and app fatigue; database-dependent estimates; variable engagement and nutritional accuracy.	Earli-er/core/contextual: [10,14,15,16,17,18,51]; 2026 update: [25,26,27,28,29,30,31,35,37,41,42]
Wearable sensor-based systems	Accelerometers, gyroscopes, acoustic sensors and ingestion-detection algorithms	Passive detection of eating events; reduced reliance on self-report	Food type and quantity usually unresolved; false positives; comfort, battery and privacy constraints.	Earli-er/core/contextual: [7,8,9,46,47,48,54]; 2026 update: none
Hybrid AI-assisted platforms	Image recognition combined with user confirmation and nutrient databases	Improved completeness compared with single-method tools; flexible user workflows	Continued user correction; error propagation across recognition, portion and database components; increased complexity.	Earli-er/core/contextual: [5,44,45,48]; 2026 update: [25,26,27,28,29,34,35,37,39]
Precision-nutrition software	Machine learning, multi-source data integration and predictive modelling	Personalised dietary recommendations; integration of metabolic and behavioural data	Limited clinical-outcome validation; model opacity and context sensitivity; high data, governance, version-control and interoperability requirements.	Earli-er/core/contextual: [18,47,49,53,54,55]; 2026 update: [34,39,40,42]

### 3.2. User Engagement, Adherence and Behavioural Barriers

Sustained user participation is a central determinant of the real-world value of dietary software. Across the literature, engagement commonly declines over time, leading to incomplete and potentially biased dietary datasets.

#### 3.2.1. Patterns of Adoption and Attrition

Dietary applications often show high initial uptake followed by declining use, but attrition patterns vary according to intervention design, user group and follow-up duration [14,15,16,17]. The difficulty of sustaining engagement shows moderate within-map convergence; a universal fixed-time attrition pattern is not justified. Partial automation can reduce reporting time under defined conditions [35], but it does not, by itself, establish sustained adherence.

#### 3.2.2. Manual Burden and Correction Fatigue

Even AI-assisted tools may require users to verify detected foods, adjust portions, provide cooking details or enter missing ingredients, particularly for mixed dishes and culturally specific foods. Earlier/core evidence describes this correction burden [14,15,16,18], while 2026-update studies provide additional usability and workflow evidence [26,28,29,30,31,35,37,39]. The cumulative work of identifying and correcting errors can be time-consuming and cognitively demanding. User-centred studies confirm that images may aid memory, while food identification and portion estimation remain burdensome [35,37].

#### 3.2.3. App Fatigue and Declining Behavioural Impact

Behavioural studies and app-usage reviews describe declining motivation, perceived usefulness and engagement over time [14,15]. Digitalisation does not remove the biases of self-reported dietary assessment; users may still omit foods intentionally or unintentionally, especially when items are difficult or inconvenient to log. This pattern shows moderate within-map convergence across the selected heterogeneous app studies.

#### 3.2.4. Effectiveness of Engagement Strategies

Many dietary applications use reminders, goal setting, gamification and social support. These strategies may improve short-term participation, but evidence for durable effects is inconsistent [15,17]. Automatic image recognition can improve reporting speed and identification accuracy under defined meal scenarios [35], while qualitative evidence shows that ease of use and image support influence preference [37]. Engagement features should therefore be evaluated as parts of the full workflow rather than assumed to solve attrition independently.

#### 3.2.5. Differential Engagement Across User Groups

Engagement differs across demographic, clinical and digital-literacy groups [15,16,17]. Users may lack the capability to correct entries, the opportunity to log consistently in real-world contexts or the motivation to continue when effort exceeds perceived benefit. These observations are compatible with the Capability, Opportunity, and Motivation–Behaviour framework, but subgroup-specific estimates remain heterogeneous. User behaviour therefore remains an important implementation factor alongside algorithmic performance.

### 3.3. Food-Composition Databases, Nutrient Accuracy and Cultural Bias

Food-composition databases form the analytical backbone of dietary software by converting logged or detected foods into estimates of energy and nutrient intake. The reviewed evidence indicates that database quality is a major determinant of software accuracy, regardless of interface design or AI-recognition performance.

#### 3.3.1. Accuracy of Energy and Macronutrient Estimation

Earlier/core evidence indicates that some digital tools achieve reasonable group-level agreement with reference methods for total energy and macronutrient estimates under defined conditions [10,16,43,44,51], with additional validation evidence in the 2026 update [25,26,27,28,29,38,39,41]. Controlled-feeding studies may show small average bias for selected methods, but wide individual limits of agreement and differential nutrient accuracy remain common [25]. One 2026 study comparing an AI-powered image app with doubly labelled water reported 25% underestimation and poor individual-level agreement in females with obesity [42]. That finding is illustrative of the broader concern about individual-level validity but is not generalisable beyond the evaluated application and population. The overall conclusion therefore shows moderate within-map convergence and remains tool-, setting- and population-dependent rather than universally “accurate”. The principal study-level validation populations or test sets, sample sizes, reference methods and key agreement results are provided in Supplementary Table S4.

#### 3.3.2. Micronutrient Estimation Errors

Micronutrient estimates are less consistent than total energy and broad macronutrient estimates in earlier/core reviews and evaluations [10,16,43,51], and in 2026-update validation studies [25,26,27,28,29,39]. Incomplete nutrient profiles, averaged composition values, limited representation of fortified products and insufficient detail on brands, cooking state or preparation methods can increase error. Geographic provenance adds another layer of uncertainty: food-composition values for minerals can vary spatially, and databases may borrow values across regions or lack metadata on where foods were sampled [56,57]. International sourcing and trade can therefore make a locally coded food entry an imperfect proxy for the nutrient composition of the item actually consumed. The direction of concern shows moderate within-map convergence, but nutrient-specific performance cannot be generalised without tool-, food-source- and population-specific validation.

#### 3.3.3. Crowdsourcing, Data Quality and Standardisation

Large and user-generated food databases can expand coverage but may contain incomplete nutrient fields, inconsistent portion units, or duplicate and misclassified entries [16,18,51]. Such inconsistencies can propagate into nutrient estimates and reduce reproducibility. Because database architecture and curation differ substantially between platforms, this limitation shows moderate within-map convergence at the field level and requires application-specific assessment.

#### 3.3.4. Cultural Bias and Global Applicability

Direct evidence of cultural under-representation remains limited/emerging within the selected evidence map rather than uniformly quantified. Earlier/regional evidence includes work in African food contexts [13], while 2026-update studies cover Malaysian, Thai and Taiwanese foods and multimodal cultural prompting [31,34,36,39]. Earlier contextual scoping reviews [44,45,49] and a 2026-update review [40] support the broader concern and identify gaps in real-world clinical applications. The magnitude of bias varies by dataset, cuisine, task and software platform.

#### 3.3.5. Consequences for Equity and Research Validity

Direct comparative dietary-software evidence indicates that database and recognition errors can differ across cuisines and preparation contexts. Earlier evidence includes cross-cultural camera work [13], and 2026-update studies add regional validation and multimodal analyses [31,34,36,39]. The earlier contextual responsible-AI literature identifies potential consequences for fairness and external validity [20,49]. Equity implications therefore remain limited/emerging within the selected evidence map. Future studies should report performance by cuisine, demographic group and preparation context rather than extrapolating from narrow image collections. Table 4 summarises the main nutrient-estimation and cultural-applicability findings.

**Table 4:** Accuracy of nutrient estimation and cultural bias in digital dietary assessment software, with key supporting references.

Assessment Dimension	Reported Accuracy Level	Evidence Summary	Implications for Research and Clinical Practice	Key Supporting References
Total energy intake	Moderate and tool-dependent	Selected methods show reasonable group-level agreement under controlled conditions, but individual error can be wide; free-living clinical performance is less consistent.	Useful for broad monitoring only after tool- and population-specific validation; not a universal claim of accuracy.	Earli-er/core/contextual: [10,16,43,44,51]; 2026 update: [25,26,27,28,29,38,39,41,42]
Macronutrients (carbohydrate, protein and fat)	Moderate and tool-dependent	Agreement is often better than for micronutrients but varies by application, database and recording workflow.	May support general pattern assessment; nutrient-specific therapeutic use requires independent validation.	Earlier/core/contextual: [10,43,44,51]; 2026 update: [25,26,27,28,29,38,39,41,42]
Micronutrients (vitamins and minerals)	Moderate within-map convergence on nutrient-specific variability	Reviews and validation studies report greater variability because of incomplete nutrient profiles, averaging, missing preparation/brand details and geographic mismatch between consumed foods and composition data.	Tool-level validation is required before deficiency assessment or therapeutic prescription; food provenance and geographic suitability of composition data should be documented.	Earlier/core/contextual: [10,16,43,51,56,57]; 2026 update: [25,26,27,28,29,39]
Fatty acids and added sugars	Persistent image-based limitation	Hidden oils, sauces and sugars may be missed by image-only or incomplete manual workflows.	Risk of underestimation should be communicated and corrected through user or professional confirmation.	Earli-er/core/contextual: [6,11,12,24,43,44,45]; 2026 up-date: [30,34,36,39]
Mixed and composite dishes	Higher within-map convergence for technical limitation	Ingredient disaggregation and portion estimation remain difficult across heterogeneous dishes and preparation methods.	Major source of uncertainty in free-living assessment.	Earli-er/core/contextual: [6,11,12,24,43,44,45]; 2026 up-date: [30,34,36,39]
Culturally diverse and traditional foods	Limited/emerging within-map convergence	Direct cross-cultural validation is limited, but studies in under-represented populations and recent reviews identify dataset and database gaps.	Performance should be reported by cuisine and population; universal generalisation is not justified.	Earli-er/core/contextual: [13,44,45,49,58]; 2026 update: [31,34,36,39,40]

### 3.4. Clinical Integration, Interpretability and Professional Trust

Although dietary software is widely used in consumer wellness settings, its integration into clinical nutrition practice remains limited. Key barriers include insufficient clinical validation, limited interpretability, unclear accountability, weak interoperability and concerns about privacy and governance.

#### 3.4.1. Clinical Validation and Evidence Gaps

Clinical readiness depends on the intended use. Earlier/core reviews and evaluations are more favourable for low-risk self-monitoring, research screening and dietitian-assisted data collection than for stand-alone therapeutic decisions [10,18,43,44,45,49,51], and 2026-update studies add controlled, app-level and multimodal validation evidence [25,26,27,28,29,38,39,41,42]. Most direct evaluations are short-term, use controlled meals or involve generally healthy participants. One physiological validation of SNAQ against doubly labelled water identified substantial bias and poor individual agreement in females with obesity [42]; this single application/population illustrates a potential high-stakes validity problem but does not establish the magnitude of error for other tools or populations. Evidence remains insufficient for nutrient-specific decisions in chronic kidney disease, diabetes, malnutrition and other settings requiring precise intake estimates.

#### 3.4.2. Explainability and Algorithmic Transparency

A major barrier to clinical trust is limited algorithmic transparency. Direct 2026-update multimodal validation identified errors related to food visibility, portion estimation, prompt construction, omission and hallucination [39], while cross-source evaluation showed that estimates varied with the contextual information supplied to the model [38]. In one separate application and population, physiological validation demonstrated substantial downstream energy-estimation bias without isolating every underlying algorithmic cause [42]; this finding is illustrative rather than generalisable to all AI dietary tools. The earlier contextual healthcare–AI literature indicates that insufficient explainability can weaken auditability, accountability and professional trust [19,20,53]. Explainability and uncertainty reporting are therefore important requirements for high-stakes use.

#### 3.4.3. Risk of Inappropriate Dietary Advice

Unsupervised use of consumer-facing dietary applications may create safety concerns when generic advice is interpreted as individual treatment. This concern is particularly relevant when the system lacks comorbidity, laboratory, medication or treatment-goal information [18,19,53]. The evidence supports professional oversight for high-risk use, while understanding that lower-risk self-monitoring may require a different threshold of validation.

#### 3.4.4. Professional Perceptions and Adoption Barriers

Clinicians may value automated dietary assessment for efficiency while remaining cautious about data quality, transparency and responsibility for errors [18,19,20,21,53]. Limited interoperability with electronic health records also restricts integration. Standards-based interfaces can improve data exchange, but they do not, by themselves, validate the dietary estimates being transferred [21].

#### 3.4.5. Privacy, Governance and Ethical Constraints

Dietary information may be combined with wearable, behavioural, metabolic or genomic data, increasing concerns about informed consent, data ownership, third-party sharing, security and long-term storage [20,21,47,53]. Institutional adoption requires clear governance and auditable data flows. Alignment with recognised interoperability standards may facilitate integration, but privacy-by-design and purpose-specific validation remain necessary. The proposed use-case framework is summarised in Table 5.

### 3.5. Wearable Sensors, Big Data Integration and Precision Nutrition

Dietary software increasingly extends beyond manual and image-based logging toward sensor-enabled and data-integrated systems. These approaches aim to reduce reliance on user input, increase temporal resolution and support personalised nutrition. The available evidence nevertheless indicates substantial technical and evidentiary limitations.

#### 3.5.1. Wearable Sensors for Passive Dietary Monitoring

Wearable sensors can detect eating-related behaviours, such as hand-to-mouth gestures, chewing and swallowing [7,8,9,46,47,48,54]. Evidence is moderately supportive for identifying eating occasions under controlled or semi-controlled conditions. Specificity decreases in free-living contexts because talking, walking, repetitive hand movements, ambient noise and motion artefacts can produce false detections [46,47,48,54]. Wearables are therefore better at indicating when eating may occur than determining exactly what and how much was consumed.

#### 3.5.2. Integration with Dietary Software Platforms

Platforms can combine sensor-detected eating events with prompts, manual logging or image capture [47,48]. This hybrid approach may reduce missed eating occasions but still requires user confirmation. Interoperability remains fragmented because devices differ in data formats, sampling rates and synchronisation protocols.

#### 3.5.3. Big Data and Multi-Source Dietary Profiling

Dietary platforms increasingly integrate activity, glucose, electronic-health-record and other data streams to construct broader nutritional phenotypes [21,47,54,55]. These approaches offer potential for richer phenotyping, but reliable multi-source integration requires harmonised food terminology, timestamps, portion definitions and data-quality controls. Increased data volume does not compensate for systematic measurement error.

#### 3.5.4. Precision Nutrition and Predictive Algorithms

Precision nutrition combines dietary data with metabolic, genomic, microbiome or glycaemic-response information to generate individualised predictions [18,47,49,54,55]. Evidence supports personalised prediction in selected settings, but sustained clinical benefit from dietary-assessment software remains insufficiently established. Short-term feasibility or surrogate outcomes should not be interpreted as proof of long-term effectiveness.

#### 3.5.5. Privacy, Governance and Scalability

The integration of dietary, behavioural and biological data creates substantial privacy, cost and governance challenges [20,21,47]. Platforms vary in consent processes, security standards and third-party data-sharing practices. Sensor-based and multi-source systems should therefore be viewed as complements to established assessment rather than universal replacements.

## 4. Discussion

This structured narrative review synthesises direct evidence while distinguishing the contextual and methodological literature. The mapped evidence base is organisational rather than exhaustive. Across the synthesis, food-recognition performance is more mature than portion estimation, nutrient quantification, sustained engagement and clinical-outcome validation. Evidence strength varies substantially by intended use, target population and reference standard.

### 4.1. Technical Advances and Persistent Measurement Error

**Figure 2:**
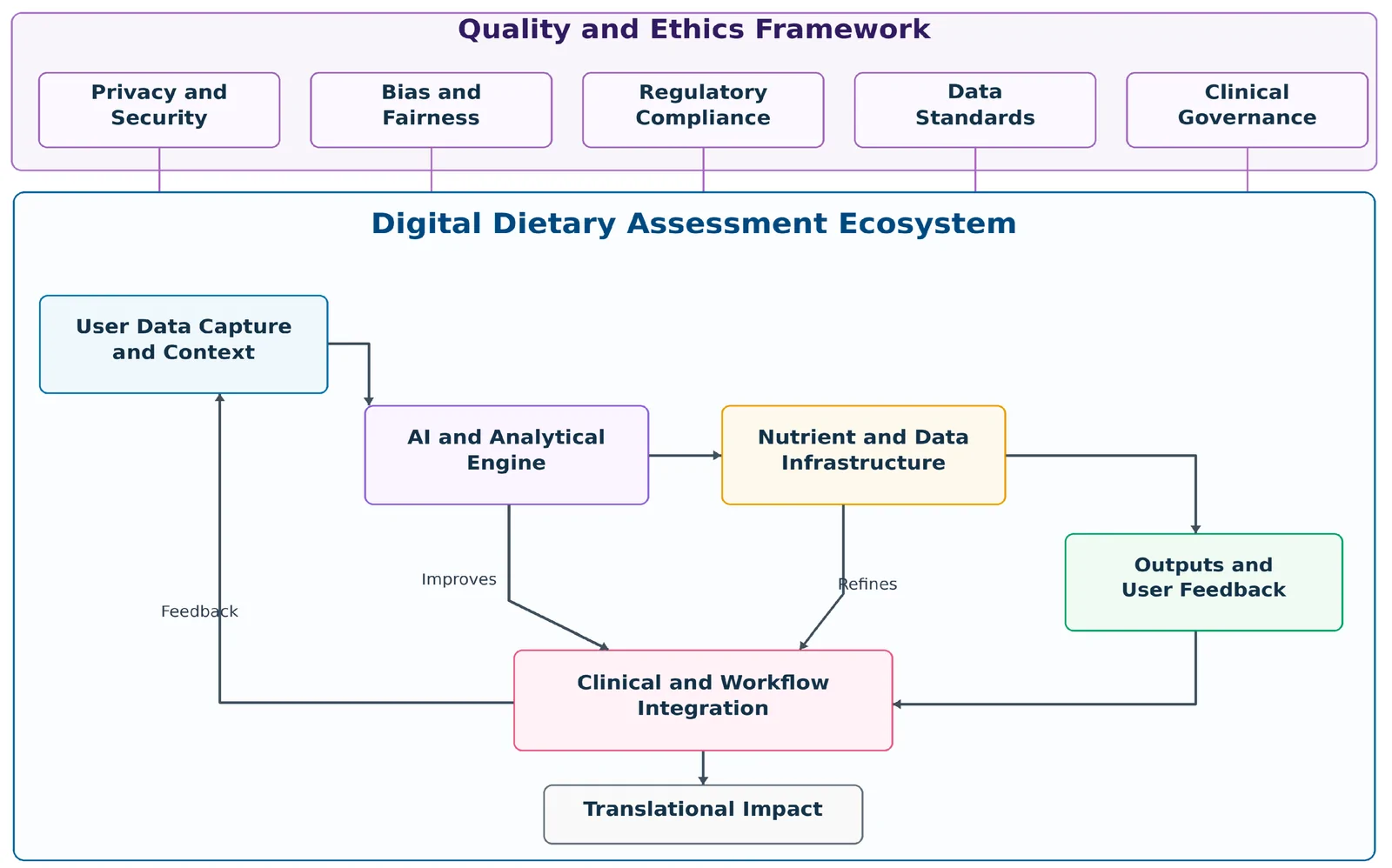
Descriptive ecosystem map of the current digital dietary-assessment workflow, showing interactions among user data capture, AI processing, nutrient infrastructure, outputs, clinical workflow integration and governance.

A central finding is that greater computational sophistication has not consistently translated into more reliable dietary measurement. Earlier/core studies show that image-recognition systems perform best for simple foods under controlled conditions [6,11,12,24,43,44,45], while 2026-update publications extend this observation to newer image and multimodal systems [30,34,35,36,38,39]. Figure 2 illustrates how user input, AI processing, nutrient databases, governance structures and clinical workflows interact. The hidden-ingredient problem further shows why improved classification alone cannot resolve the full measurement task.

### 4.2. Data Density and Systematic Measurement Bias

Digital tools can generate frequent observations, but repeated measurement does not eliminate systematic bias when the same errors recur [1,2,10,44,45]. Underreporting, selective logging and incomplete correction can persist despite automation. One physiological validation against doubly labelled water found substantial systematic underestimation and poor individual agreement for a specific AI-powered image app in females with obesity [42]. This study illustrates that data density and computational sophistication do not, by themselves, guarantee validity; its effect size should not be generalised to other applications or populations. High data density may therefore increase the number of biased observations without necessarily improving validity. Multimodal approaches may reduce selected errors, but they require independent nutrition-specific validation.

### 4.3. Behavioural Constraints and Adherence

Human behaviour remains an important determinant of digital dietary-assessment quality. Sustained adherence is difficult across many platforms, although attrition is heterogeneous and should not be expressed as a universal fixed-time pattern [14,15,16,17]. Direct usability and qualitative studies show that automation can improve reporting speed and that food images can support recall, while users may still face correction, portion-estimation and workflow burdens [35,37]. Behavioural features may support engagement but do not remove the burden of intensive self-monitoring.

### 4.4. Cultural Applicability and Equity

Direct dietary-software evidence of cross-cultural performance remains limited. Earlier evidence includes African food-context work [13], while 2026-update studies add Malaysian, Thai, Taiwanese and multimodal cultural-context evidence [31,34,36,39]. Earlier contextual reviews and responsible-AI literature [20,44,45,49] and a 2026-update scoping review [40] identify broader risks for fairness and global generalisability. The concern is scientifically justified, but the magnitude of cross-cultural bias should be measured by cuisine, task and population rather than assumed.

### 4.5. Clinical Trust, Explainability and Professional Accountability

Limited clinical uptake is associated with concerns about transparency, accountability and safety. Direct 2026-update multimodal validation shows that food visibility, portion estimation, prompting, omission and model output can produce clinically relevant error [39]. App-level comparison and physiological validation separately demonstrate substantial output variability and bias [41,42]; however, the latter is a single application/population and should not be interpreted as a universal estimate of error. Earlier contextual healthcare–AI literature supports the need for auditable explanations, governance and professional accountability [19,20,53]. Explainability, uncertainty reporting and clinician-facing audit features should therefore be prioritised for high-stakes applications.

### 4.6. Precision Nutrition: Current Evidence and Limitations

The integration of dietary software with wearables, biomarkers and multi-omics data has created enthusiasm for precision nutrition [18,47,49,54,55]. While 2026-update multimodal studies demonstrate food-recognition and context-aware capabilities [34,39], one recent physiological validation in females with obesity illustrates that technical sophistication in a specific application does not guarantee individual-level dietary validity [42]. This single study is cautionary rather than generalisable. Feasibility, technical benchmark performance and clinical-outcome evidence should therefore be reported separately.

### 4.7. Implications for Future Development and Research

Future progress should prioritise methodological robustness over technological novelty. Validation requirements should be explicitly matched to use case. Consumer self-monitoring may tolerate broader error bounds than longitudinal epidemiology, while individual therapeutic decisions require nutrient-specific accuracy, uncertainty reporting, independent validation and professional oversight. Additional priorities include passive or semi-passive sensing; culturally inclusive databases; explainable AI; interoperable data standards; and governance models addressing consent, privacy and ownership.

Future validation studies should report more than aggregate correlation or overall classification accuracy. Useful reporting would include food- and nutrient-specific error distributions, calibration across demographic and cultural groups, performance in mixed dishes, rates of user correction, missing-event frequency, and stability across devices and software updates. Studies intended to support clinical adoption should prospectively define the intended decision, acceptable error range and consequences of misclassification. They should also compare automated output with an appropriate reference method under free-living conditions and describe how uncertainty is communicated to users and clinicians. These requirements would make it possible to distinguish incremental technical improvement from evidence that a tool is safe and useful for a specified nutritional task.

## 5. Prospective Directions and Conceptual Framework for Clinically Reliable Digital Dietary Assessment

The evidence indicates that future dietary-assessment systems should address persistent limitations in accuracy, adherence, cultural inclusiveness and clinical trust. Progress depends on how systems are designed, validated and integrated into healthcare. Based on the synthesis, this review proposes a conceptual framework for clinical reliability and broader applicability.

### 5.1. From Active Logging to Passive Dietary Sensing

Current dietary software continues to rely heavily on active user participation. Even AI-assisted tools require users to verify outputs, correct errors and complete missing information. Acoustic, motion-based and other wearable approaches may help identify eating occasions with lower user burden [7,8,9,46,47,48,54]. Because sensors alone cannot reliably determine food type and quantity, future systems should combine passive detection with concise confirmation workflows.

### 5.2. Explainable Artificial Intelligence as a Clinical Requirement

For clinical adoption, computational transparency must be embedded into system design. Explainable-AI frameworks can help clinicians trace how estimates are generated, identify uncertainty and evaluate reliability at the individual level [19,20,53]. Practical features should separate errors arising from recognition, portion estimation, database limitations and user correction, and they should permit clinician audit and override.

### 5.3. Culturally Inclusive and Region-Specific Food Databases

Food databases should move beyond narrow reference systems toward modular and regionally validated structures. Future software should incorporate local ingredients and preparation methods, region-specific food ontologies and validation datasets that represent culturally diverse meals. Evidence supporting the need for such work remains limited/emerging within the selected evidence map and should be strengthened through direct comparative studies [13,44,45,49,58].

### 5.4. Standardised Validation Pathways for Clinical Use

Validation practices remain fragmented and are often poorly aligned with intended clinical use. Tools intended for medical decision support should be benchmarked against accepted reference methods and evaluated separately for energy, macronutrients, micronutrients and other clinically important dietary components [10,25,26,27,28,29,39,42,43,44,45,51]. Validation should include free-living conditions, longitudinal follow-up, software-version documentation and the populations in which the tool will be used.

### 5.5. Ethical Regulation, Privacy and Data Stewardship

As dietary software integrates behavioural, physiological and biological data, ethical regulation becomes central to scalability and trust. Future systems should adopt privacy-by-design, clarify ownership and third-party access, and give users meaningful control over their information [20,21,47,53]. Platforms intended for clinical use should demonstrate data-security safeguards and transparent governance.

### 5.6. Proposed Conceptual Framework

**Figure 3:**
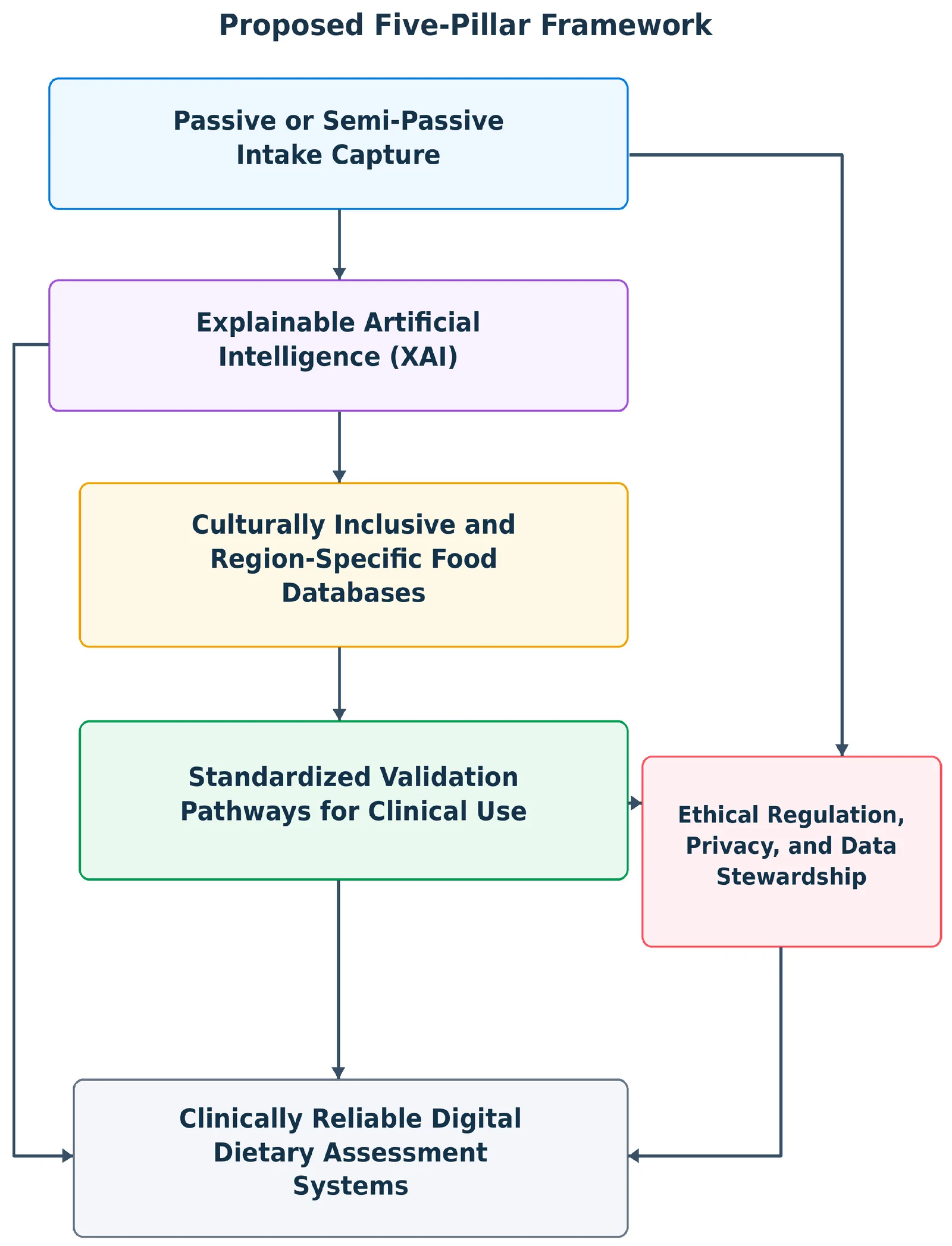
Proposed future-facing five-pillar framework for clinically reliable digital dietary-assessment systems. This figure summarises the validation, data, behavioural, clinical-integration and governance requirements proposed for clinical readiness.

Figure 3 summarises the proposed five-pillar framework. A clinically reliable dietary-assessment system should integrate five interdependent pillars: passive or semi-passive intake capture, explainable artificial intelligence, culturally inclusive and region-specific food databases, standardised validation pathways for clinical use and ethical regulation, privacy and data stewardship. Multidisciplinary collaboration is necessary. Behavioural scientists can help design sustainable engagement workflows; data engineers can build interoperable and explainable architectures; clinical nutrition experts can define validation endpoints; and ethicists can shape responsible governance. Reliability depends on the integration of technical accuracy, human-centred design and clinical accountability rather than on any single technological advance.

## 6. Conclusions

Dietary software has evolved from basic digital food logs into AI-assisted systems combining computer vision, wearable sensing and multimodal foundation models. Within the purposively selected evidence map, higher within-map convergence is observed for selected food-recognition tasks and for the persistence of some technical limitations, moderate within-map convergence characterises energy/macronutrient validity and engagement-related conclusions, and limited/emerging within-map convergence remains for culturally diverse performance and stand-alone clinical readiness. These descriptors summarise convergence within the selected literature only; they do not indicate completeness of retrieval, formal certainty of evidence or reproducibility of screening. Multimodal studies demonstrate technical gains under defined conditions, while physiological validation shows that substantial error can persist in free-living clinical populations.

Readiness must be judged by intended use. Current tools may support consumer self-monitoring, research screening and dietitian-assisted assessment when their limitations are understood and outputs can be reviewed. The evidence does not support stand-alone use for precise sodium, potassium, protein or micronutrient prescription, or for other high-stakes individual therapeutic decisions without independent validation in the target population and professional oversight.

Future work should prioritise externally validated systems that combine low-burden intake capture with culturally inclusive food databases, transparent uncertainty reporting, standardised nutrient-specific validation and clear privacy governance. Until such evidence is available, AI-enabled dietary-assessment software should be considered complementary to, rather than a replacement for, established dietetic assessment and professional judgement.

## Figures and Tables

**Table 1:** Structured literature-search and evidence-mapping workflow.

Stage	Purpose	Decision or Output
Scope definition	Define review question, technologies, populations, outcomes and intended uses.	Purposively selected structured narrative-review scope established.
Primary search	Search five electronic sources for literature published mainly from January 2020 to March 2025.	Retained aggregate review-log totals: 1620 electronic-source records + 42 additional records = 1662 before deduplication; 478 duplicates removed; 1184 screened; 972 excluded at title/abstract stage; 212 full texts assessed; 197 excluded; 15 sources retained. Database-level allocation, exact historical execution dates and itemised full-text exclusion reasons were not retained at record level; Figure 1 therefore presents a best-available aggregate reconstruction rather than a fully reconstructable PRISMA audit trail.
2026 literature update	Rerun all five electronic sources across January 2020–7 August 2026 and repeat backward/forward citation tracking on 7 August 2026. Historical-period recovery/reassessment was handled separately from the post-March-2025 update-specific screening stream. MEDLINE-indexed records were retrieved within PubMed; no separate MEDLINE search was performed.	2146 electronic-source records + 73 citation-tracking records = 2219 identified; 69 duplicates removed = 2150 unique records. Historical pathway: 1653 January 2020–March 2025 records were removed from update-specific repeat screening; 10 historical publications [25,26,27,28,29,30,31,32,33,34] were recovered/reassessed separately. The retained audit trail does not preserve a study-level electronic-source-versus-citation or earlier-EndNote-library split for those 10 publications; no route-specific allocation was reconstructed. Post-March-2025 pathway: 497 screened; 402 excluded at title/abstract stage; 95 full texts assessed; 87 excluded; 8 publications [35,36,37,38,39,40,41,42] incorporated. Total update publications = 18. See Supplementary Table S1A.
Eligibility assessment	Assess direct relevance, peer-review status, methodological transparency, English language and contribution to the review question.	Sources classified as direct, contextual, methodological or not used.
Direct-evidence mapping	Record design, population, technology, outcome, reference standard, limitations and overlap.	Direct validation and secondary evidence kept distinct.
Contextual/methodological mapping	Record implementation, governance and reporting sources separately.	Contextual literature does not count as direct confirmation.
Conclusion appraisal	Compare directness, convergence, reference standards, limitations and overlapping evidence.	Principal conclusions described using the Section 2.7 within-map convergence descriptors (higher, moderate or limited/emerging); these are descriptive narrative heuristics, not formal certainty grades. Overlapping review-of-review evidence is not counted as independent primary replication. Detailed in Supplementary Table S3.

**Table 5:** Proposed decision framework for evaluating the clinical readiness of digital dietary-assessment software.

Intended Use	Current Evidence-Based Readiness	Main Limitations in Current Evidence	Author-Proposed Validation Standard
Consumer self-monitoring	Conditionally suitable for low-risk awareness and trend tracking.	Tool-dependent accuracy; omission, portion and database error; advice may be mistaken for treatment.	Transparent uncertainty and limitations; broad usability testing; no unqualified medical claims.
Dietitian-assisted dietary recall	Potentially suitable as an adjunct when outputs can be reviewed and corrected.	Human correction reduces but does not eliminate recognition, portion and database errors.	Comparison with an accepted reference method; auditable corrections; clinician review and override.
Research screening or population surveillance	Potentially suitable for defined questions after calibration.	Group-level performance may conceal individual error; differential bias across populations.	Stable group-level calibration, version control, population-specific validation and measurement-error analysis.
Longitudinal epidemiology	Not established as a universal stand-alone method.	Attrition, software updates and persistent systematic error may distort exposure–outcome associations.	Long-term adherence, version stability, reproducibility and quantified systematic error across subgroups.
Individual therapeutic decisions	Not ready for stand-alone high-stakes use.	Limited target-population validation; nutrient-specific error; uncertain accountability and safety.	Nutrient-specific validation in the target clinical population, prespecified acceptable error, uncertainty reporting and accountable professional oversight.

## Data Availability

The evidence analysed in this review is contained in the cited publications. The updated supplementary bibliographic/provenance workbook provides the validated 58-reference register, search-strategy and eligibility documentation, retained aggregate screening-flow information, and current-source verification for the 10 historical-period update publications. The separate included-evidence workbook provides a plain overview of the 33 evidence-map publications.

## Supplementary Materials

The following supporting information can be downloaded at https://www.mdpi.com/article/10.3390/nu18203406/s1. The publications listed in both supplementary workbooks correspond to References [1,2,3,4,5,6,7,8,9,10,11,12,13,14,15,16,17,18,19,20,21,22,23,24,25,26,27,28,29,30,31,32,33,34,35,36,37,38,39,40,41,42,43,44,45,46,47,48,49,50,51,52,53,54,55,56,57,58] in the main manuscript and are already cited in the article. The final bibliographic/provenance workbook provides the preserved search expressions and source scope (Supplementary Table S1); the update screening-flow summary (Supplementary Table S1A); current retrievability and unresolved historical entry routes [25,26,27,28,29,30,31,32,33,34] (Supplementary Table S1B); operational eligibility criteria (Supplementary Table S1C); the validated article register and the included-evidence map (Supplementary Table S2); the conclusion-level higher/moderate/limited–emerging within-map convergence table (Supplementary Table S3); and the principal original quantitative validation-study table with populations or test sets, sample sizes, reference methods, key accuracy/agreement results and verified secondary-review overlap (Supplementary Table S4). A separate plain included-evidence workbook lists the currently mapped 33 publications and records Cofre et al. [25] as a later post-primary secondary-synthesis addition rather than a primary-phase source. The retained aggregate primary log of 15 sources is preserved separately because the unmatched fifteenth historical source identity cannot be reconstructed from the available record. The supplementary files are bibliographic and reproducibility aids and do not retrospectively create missing screening routes or inter-reviewer reproducibility statistics.
